# Pre-operative assessment of living liver donors’ liver anatomy and volumes

**DOI:** 10.1007/s13304-024-01806-6

**Published:** 2024-03-25

**Authors:** Nicolas Goldaracena, Paola A. Vargas, Lucas McCormack

**Affiliations:** 1 Department of Surgery, Division of Transplantation, University of Virginia Health System, 1215 Lee Street, PO Box 800709, Charlottesville, VA 22908-0709 USA; 2 Transplant Unit, Hospital Aleman de Buenos Aires, Buenos Aires, Argentina

**Keywords:** Living donor, Living donor liver transplantation, Preoperative assessment, Anatomic variants, Liver volumes

## Abstract

Decades of experience supports LDLT as a favorable strategy to reduce waitlist mortality. The multiple regenerative pathways of hepatocytes and other hepatic cells justify the rationale behind it. Nonetheless, living liver donation is still underused and its broader implementation is challenging, mostly due to variability in practices leading to concerns related to donor safety. A non-systematic literature search was conducted for peer-reviewed original articles related to pre-operative evaluation of living liver donor candidates. Eligible studies were synthesized upon consensus for discussion in this up-to-date review. Review of the literature demonstrate that the importance of preoperative assessment of vascular, biliary and liver volume to ensure donor safety and adequate surgical planning for graft procurement is widely recognized. Moreover, data indicates that anatomic variants in vascular and biliary systems in healthy donors are common, present in up to 50% of the population. Therefore, comprehensive mapping and visualizations of each component is needed. Different imaging modalities are reported across practices and are discussed in detail. Lastly, assessment of liver volume must take into account several technical and donor factors that increase the chances of errors in volume estimation, which occurs in up to 10% of the cases. Experience suggests that maximizing donor safety and lessening their risks is a result of integrated experience between hepatobiliary and transplant surgery, along with multidisciplinary efforts in performing a comprehensive pre-operative donor assessment. Although technical advances have increased the accuracy of volume estimation, over- or under-estimation remains a challenge that needs further attention.

## Introduction

Waitlist mortality continues to be a noxious reality despite constant efforts to reduce it. The limited availability of acceptable quality grafts from deceased donors in relation to the increasingly high demand has lead transplant teams to constantly search for alternatives to replenish the organ pool. Decades of experience supports living donor liver transplant (LDLT) as a feasible pathway to achieve faster access to transplantation, and therefore, as a favorable strategy to reduce waitlist mortality [1, 2]. In addition, the comprehensive pre-operative evaluation of living donor candidates ensures transplantation with overall high-quality allografts conveying additional benefits, including higher survival rates to the recipient when compared to LT with deceased donors [3–5].

The multiple regenerative pathways of hepatocytes and other hepatic cells justify the rationale behind LDLT. More importantly, this intrinsic capacity is even more evident within normal hepatic tissue [6], which is expected to be the case for a living donor. Experimental models have demonstrated activation of pathways leading to liver regeneration as early as 2 h after partial hepatectomies [6]. In humans, increased-regenerative markers have been observed within the first days following living donor hepatectomy, with evidence of almost all functional and volumetric recovery within the first 3–4 months up to over a year depending on the donated graft [7–9]. Although differences in the regenerative extent and time between donors and recipients have been observed, the regenerative ability of the liver also safeguards that the transplanted segment into the recipient will restore liver function and volumes, with observed reconstitution percent up to 93% after 3 months of LDLT [10–12].

Despite that, LDLT is still underused and broader implementation of this technique has been challenging [1]. In a recent meeting report by the American Society of Transplantation LDLT Consensus Conference Working Group, three main themes were identified as barriers limiting LDLT, including position toward it and data gaps related to candidate and donor selection, as well as post-transplant outcomes and long-term monitoring [1]. Interestingly, the theme with the greatest number of identified barriers was related to evaluation and selection for LDLT, which highlighted the existence of a “gray zone” in donor selection, mostly driven by the current variability in donor assessment across centers. Consequently, this variability limits the ability to quantify relative and absolute donor risk, a major pillar for LDLT [1]. Therefore, standardization of practices and a clear understanding of the pre-operative evaluation for living donors is crucial. Here, we aim to present an up-to-date review of pre-operative assessment practices of living liver donor candidates, with a particular focus on vascular and biliary, as well as volumetric evaluation.

## Methods

A non-systematic literature search was conducted in PubMed/Medline database during August–October 2023 for peer-reviewed original articles related to pre-operative evaluation of living liver donor candidates. No time limitations filters were set. However, in order present an up-to-date review, articles published in the last 5–10 years were preferentially included. English-language literature including retrospective studies, single/multicenter cohorts, case reports, as well as systematic reviews and meta-analysis were reviewed. Google Scholar database was also queried as an attempt to capture available literature in understudied topics. Articles deemed relevant were assessed and selected upon consensus among authors for discussion in this comprehensive narrative synthesis of previously published information.

## Addressing the challenges

Minimizing donor risks and complication rates is a primary goal during LDLT. Although mortality rate following living donor hepatectomy is close to zero, reported minor and major complications rates range from 1.7 to 43.1%, and 2.1 to 28%, respectively [13–16]. The wide range might reflect the variability among practices across centers and the lack of standardization when it comes to pre- and post-operative assessment of living liver donors. Previous reports have shown that increasing experience and volume of cases are associated to improved donor outcomes [14, 16]. Therefore, it is important that the transplant community—particularly those interested in performing LDLT—recognize that optimization of donor safety is a result of team effort, rather than individual work. In our experience, this goal can be achieved by establishing a multidisciplinary living donor team that continuously evaluate tasks checklists, performs weekly planning meetings and quality reviews, provides active social and ethics support, and share a commitment to quality improvement and generating new knowledge.

## Donor evaluation

Despite slight variations depending on the region of practice, criteria for living liver donation often consider candidates that are in good overall physical and mental health, are ≥ 18 years of age—and generally < 60y—, and are able to provide informed consent [17–19]. The medical evaluation must address areas related to general medical history, psychosocial history, physical exam, transmittable disease testing, case-specific and liver-related laboratory testing, and anatomic assessment [17]. As pertinent for the present review, we will now discuss the components of the anatomic assessment.

## The importance of vascular anatomy evaluation and volume estimation

Anatomic assessment of a living liver donor candidate is perhaps the most challenging, yet most important, step during pre-operative evaluation. A summary of important considerations during anatomic assessment of living liver donor candidates is depicted in Table 1. A comprehensive radiologic evaluation allows not only to ensure eligibility and quality of the potential graft, but also to perform appropriate surgical planning for both procedures, the donor hepatectomy and the recipient transplant [20, 21]. Donor candidates must complete serial assessments of vascular anatomy, biliary anatomy and liver volumes. Although, the lack of recognized guidelines limits standardization of practices and imaging modalities to be used, donor safety remains the primary goal during evaluation. Therefore, imaging techniques must be carefully selected based on each center’s resources availability and expertise [21]. Moreover, it is of utmost importance to meet the requirements set forth by designated transplant organizations depending on the region of practice to ensure that the transplant hospital has adequate resources in place to perform living donor assessment, surgical procedures and guarantee adequate recovery. For instance, detailed requirements for liver transplant programs that perform living donor recovery in the United States can be found on the Organ Procurement and Transplantation Network (OPTN) Membership Application for Liver Transplant Programs Part 9 [22].

**Table 1:** Anatomic assessment of living liver donor candidates

Benefits: Ensure eligibility and quality of the potential graft Perform appropriate surgical planning for both procedures, the donor hepatectomy and the recipient transplant					
	Vascular anatomy	Venous anatomy		Biliary anatomy	Liver volumes
	Hepatic artery	Portal vein	Hepatic veins		
Available imaging technique	Contrast-enhanced CTA with 3D reconstruction MRA with Gadolinium-based contrast agents	3D reconstruction CT with volumetric measurements MRI (provides the highest contrast resolution)		Gold standard: Intraoperative cholangiogram with cannulation of the cystic duct Preferred: MRCP	Manual techniques: calculations based on surface body area Semi-automatic: conventional CT or MRI volumetry Fully-automatic: CT-based segmentation of the liver with 3D reconstructions
Special pre-operative considerations	Careful measure of the length of the arteries before bifurcation Most common anatomic variants: Right hepatic artery passing behind or through the head of the pancreas (3.7%) Accessory LHA running along the ligamentum venosum fissure with the native LHA running a normal course. (3.2%) Exposure to high radiation Inherent risks associated with intravenous contrast use	Anatomic variations are present in up to 35% of the population Portal system variants are often associated with variants in the biliary system Common PV variations observed in living donor candidates include: Trifurcation of the main PV Caudal origin of the right posterior segmental branch Left PV origin of the right-posterior segmental branch	Anatomic variants are present in approximately 40% of living donor candidates Most common variation: Presence of an accessory (inferior) right hepatic vein draining segment VI and the inferior aspect of segment VII	Anatomic variations are more common in the right hepatic duct Common variants include: Drainage of the right-posterior duct into the left hepatic duct Trifurcation of the biliary ducts Right posterior duct draining into the common hepatic duct	Common practice is to ensure a RLV/TLV ratio ≥ 30% Factors influencing errors in volume estimation include: Technique used Donor age Donor gender Donor steatosis
Special surgical considerations	Reconstruction methods include: Arterioplasty to create a single donor artery Separate anastomoses to the right and left hepatic arteries and cystic artery End-to-side anastomosis of one donor artery to the proper hepatic artery	Reconstruction of the PV orifice depends on the type of anomaly Several methods are described, the most common are: Unification or back-wall plasty Back-wall plasty with saphenous vein graft interposition Y-graft interposition with cryopreserved iliac vein graft	In the presence of segment V and VIII tributaries with diameters ≥ 4 mm or > 5 mm reconstruction must be performed to reduce the risk of anterior segment congestion, graft dysfunction and to improve recipient outcomes	Risk factors associated with biliary complications include anatomic intrahepatic duct type, donation of the right lobe and number of bile duct orifices in the RL Due to the high technical complexity and risk for complications, donors with 3 or more right hepatic ducts, as well as those with variations that could compromise segmental bile drainage should be avoided	Imaging volume estimations can vary up to 10% from the actual graft weight
Risks of inappropriate pre-operative assessment	Inadvertent intraoperative injury Excessive dissection Graft hypoperfusion Increased risk of hepatic artery thrombosis	Poor surgical planning of parenchymal transection plane Increased risk for PV thrombosis in the recipient	Overlooking factors that affects outflow hemodynamics may result in negative postoperative outcomes, including development of SFSS	Failure to identify complex biliary configurations may result in development of biliary complications, including bile leaks and biliary fistulas, leading to important donor morbidity following living donor hepatectomy	Inadequate volume estimation can lead to hepatic dysfunction The risk of post-hepatectomy liver failure can be as high as 32% Overall and minor morbidity is higher among donors with RLV/TLV < 30%

*CTA* computed tomography angiography, *HA* hepatic artery, *MRA* magnetic resonance angiography, *MRCP* magnetic resonance cholangiopancreatography, *MRI* magnetic resonance imaging, *PV* portal vein, *RLV/TLV* remnant liver volume to total liver volume ratio, *3D* three-dimensional

### Vascular anatomy

#### Arterial anatomy

Identification of presence of hepatic artery (HA) anatomic variations, as well as adequate length measurement of the arteries before bifurcation are crucial components of the surgical planning for graft procurement and reconstruction, which can be quite challenging and complex depending on the anatomic variation. Three-dimensional (3D) reconstruction of the HA anatomy using contrast-enhanced computed tomography angiography (CTA) and magnetic resonance angiography (MRA) with Gadolinium-based contrast agents are the main imaging techniques for arterial assessment [21, 23]. In comparison with two-dimensional CT, the higher spatial resolution of 3D—CTA imaging allows visualization of even the most complex hepatic artery branches configurations, including evaluation of segment IV anatomy [21, 23, 24]. CTA potential superiority remains true against MRA, which has lower spatial resolution than CTA, is more prone to respiratory motion artifacts and is more technically demanding [21]. Therefore, CTA is usually the preferred modality across centers. Nonetheless, associated risks including exposure to high radiation and inherent risks associated with intravenous contrast use should be consider when assessing living donor candidates [25]. Recent efforts toward non-contrast modalities to minimize such harms without compromising visualization scores and diagnostic ability seems promising and need to be further evaluated [25, 26].

**Fig. 1:**
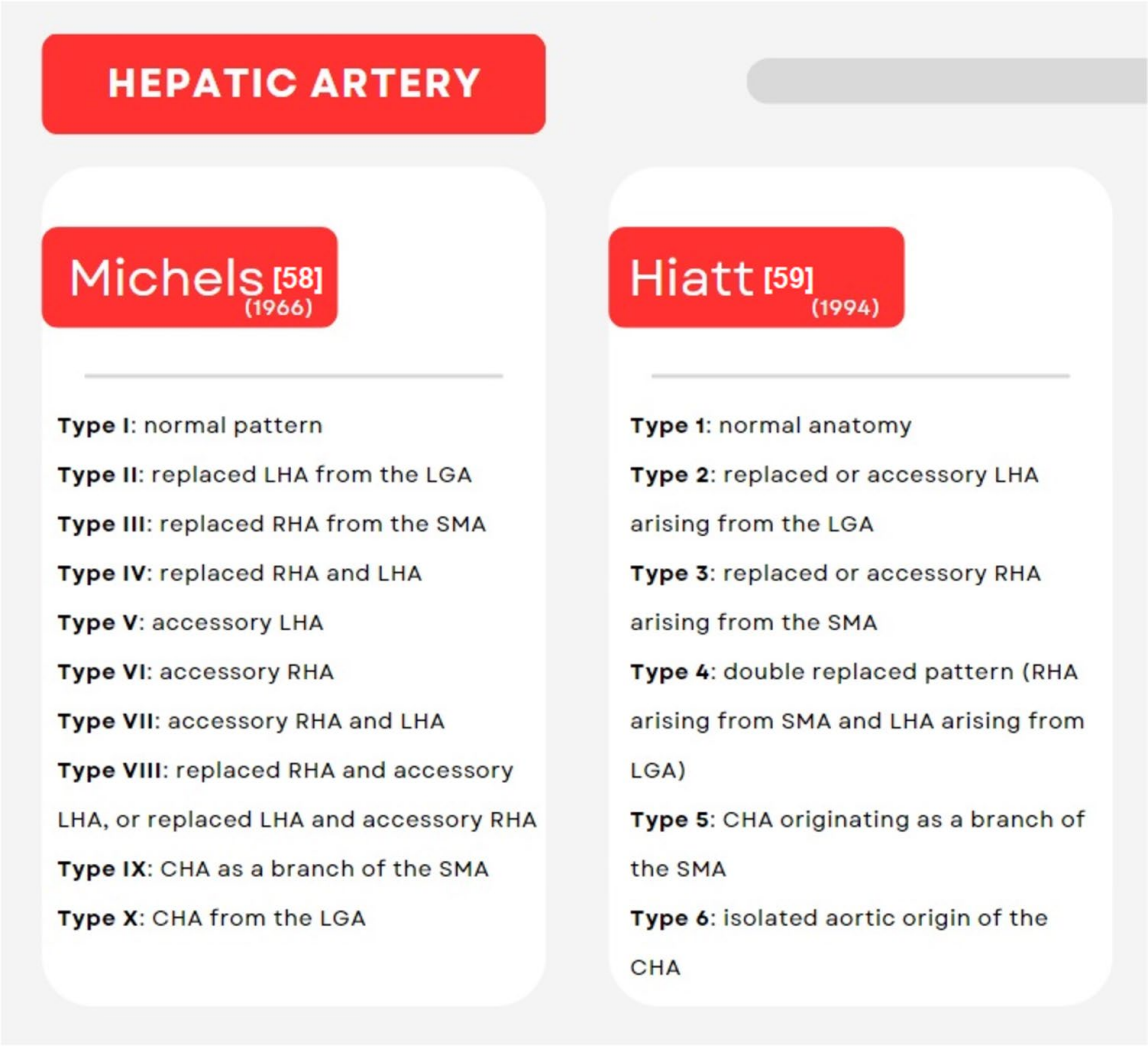
Common/clinically relevant classifications for anatomic variants of the hepatic artery. *CHA* common hepatic artery, *LGA* left gastric artery, *LHA* left hepatic artery, *RHA* right hepatic artery, *SMA* superior mesenteric artery [58, 59]

The conventional hepatic arterial anatomy, where the common hepatic artery (CHA) arises from the celiac trunk, then branches into the gastroduodenal artery and proper hepatic artery, with the latest further bifurcating into the right (RHA) and left hepatic artery (LHA), is present in about 55 to 76% of the donors [21, 24, 27]. Nevertheless, variations in intrahepatic and extrahepatic branching of hepatic arteries have been observed in up to 50% of the cases. Commonly used classifications for anatomic variants of the hepatic artery are shown in Fig. 1. Inadequate evaluation of such variants leads to detrimental peri- and post-operative complications for donors and recipients, including inadvertent intraoperative injury, excessive dissection, graft hypoperfusion, and increased risk of hepatic artery thrombosis [21, 24]. Important attention must be placed in situations where the right hepatic artery passes behind or through the head of the pancreas, and cases where an accessory LHA runs along the ligamentum venosum fissure with the native LHA running a normal course. These variations are the two most common, reported to be present in 3.7% and 3.2% of the donors, respectively [21]. Nonetheless, surgeons are required to have vast knowledge of the normal anatomy and possible variations to plan ahead complex reconstructions when needed.

#### Venous anatomy

Hemodynamics of the portal system and hepatic veins are complex. Moreover, segmental and territorial venous distribution differs vastly among individuals [28, 29]. Thus, favorable outcomes for both, donors and recipients, depend on adequate determination of volume dominance patterns, detailed venous mapping and comprehensive flow assessment [29]. The preferred imaging modalities are 3-dimensional (3D) reconstruction CT with volumetric measurements and magnetic resonance imaging (MRI) [21, 30]. MRI provides the highest contrast resolution for evaluation of venous structures depending on the sequence used (i.e., hepatobiliary phase T1-weighted and steady-state free precession) [21]. Current evidence suggest that 4-dimensional (4D) flow MRI allows for non-invasive detailed anatomic and functional assessments, including measurements of velocity and flows in the portal vein (PV) [28]. However, wide clinical implementation of 4D flow MRI is limited by its long-acquisition time and bounded availability [28].

##### Portal vein

In about 65% of the population, the PV is formed by the confluence of the splenic vein and the superior mesenteric vein, later branching into a left PV—supplying hepatic segments I to IV—and a right PV—supplying segments V and VIII anteriorly, and segments VI and VII posteriorly—[31]. On the other hand, anatomic variations are present in up to 35% of the population [21, 31]. Although, anatomic variants of the left PV rarely interfere with donor candidacy [23], variations in the right PV can confer significant risks for the remnant liver, interfere with the parenchymal transection plane, and increase the risk for PV thrombosis in the recipient [21, 30–33]. In addition, due to similar embryologic origins, portal system variants are often associated with variants in the biliary system [21, 32–34]. Thus, these cases are usually of high technical complexity and detailed pre-operative imaging assessment and planning of reconstruction techniques are needed to guarantee safe use of such grafts.

**Fig. 2:**
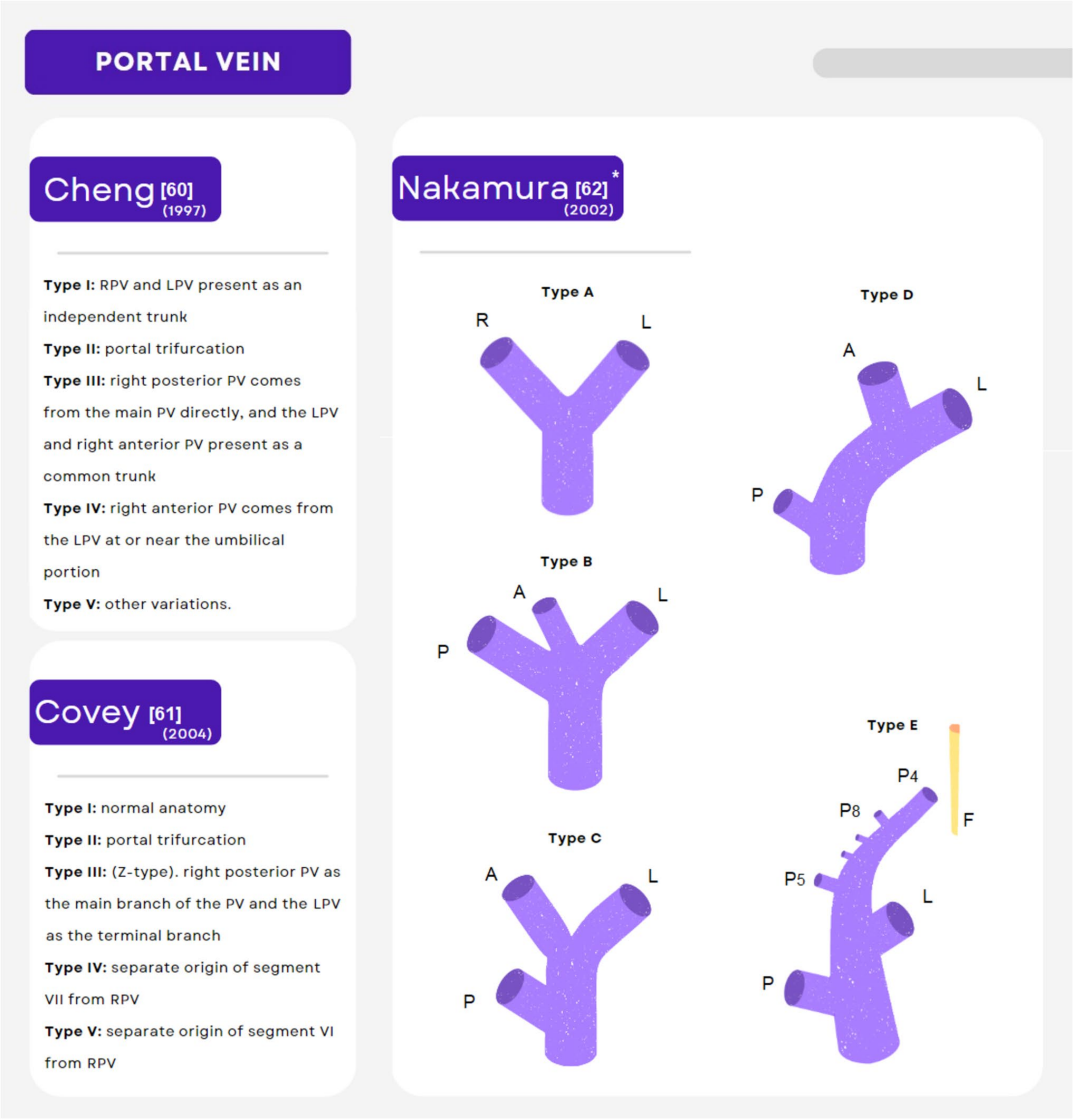
Common/clinically relevant classifications for anatomic variants of the portal vein. *A* anterior branch, *F* falciform ligament, *LPV* left portal vein, *P* posterior branch, *P*_*4*_ portal venous branch to segment 4, *P*_*5*_ portal venous branch to segment 5, *P*_*8*_ portal venous branch to segment 8, *PV* portal vein, *RPV* right portal vein; *Anatomic variations of the intrahepatic portal vein [60–62]

Common PV variations observed in living donor candidates include trifurcation of the main PV, caudal origin of the right-posterior segmental branch, and left PV origin of the right-posterior segmental branch [30, 31, 33] (Fig. 2). In the conventional presentation, the donor’s right PV is usually anastomosed to the recipient’s main portal trunk [30]. In the presence of anatomic variations, reconstruction of the PV orifice depends on the type of anomaly and can be achieved by unification or back-wall plasty, back-wall plasty with saphenous vein graft interposition, or Y-graft interposition with cryopreserved iliac vein graft, among others. More complex scenarios may require reconstruction using quilt venoplasty and vessel extension using cryopreserved iliac veins [30, 32]. Yilmaz and colleagues described the Malatya approach for reconstruction of anomalous PV configurations in the right lobe for LDLT and found significantly less rates of PV thrombosis and longer survival rates when compared to traditional reconstruction techniques [30]. Decision on which reconstruction technique to perform should be made on a case-by-case basis and requires strict follow-up of PV patency, including intraoperative Doppler ultrasonography and sequential post-operative follow-up according to each center’s protocol. Detailed surgical description of the different available reconstruction techniques and grafts, as well their advantages/disadvantages have been previously discussed elsewhere [30, 32, 33, 35].

##### Hepatic veins

The impact of venous outflow in graft regeneration and its role in postoperative outcomes is well elucidated [21, 35]. Important considerations when evaluating donor’s hepatic veins (HV) for appropriate surgical planning and determination of recipient’s adequacy include: (1) anatomy of HV, particularly middle hepatic vein (MHV) and venous tributaries, (2) recipients’ risk factors, and (3) donors’ risk factors.

**Fig. 3:**
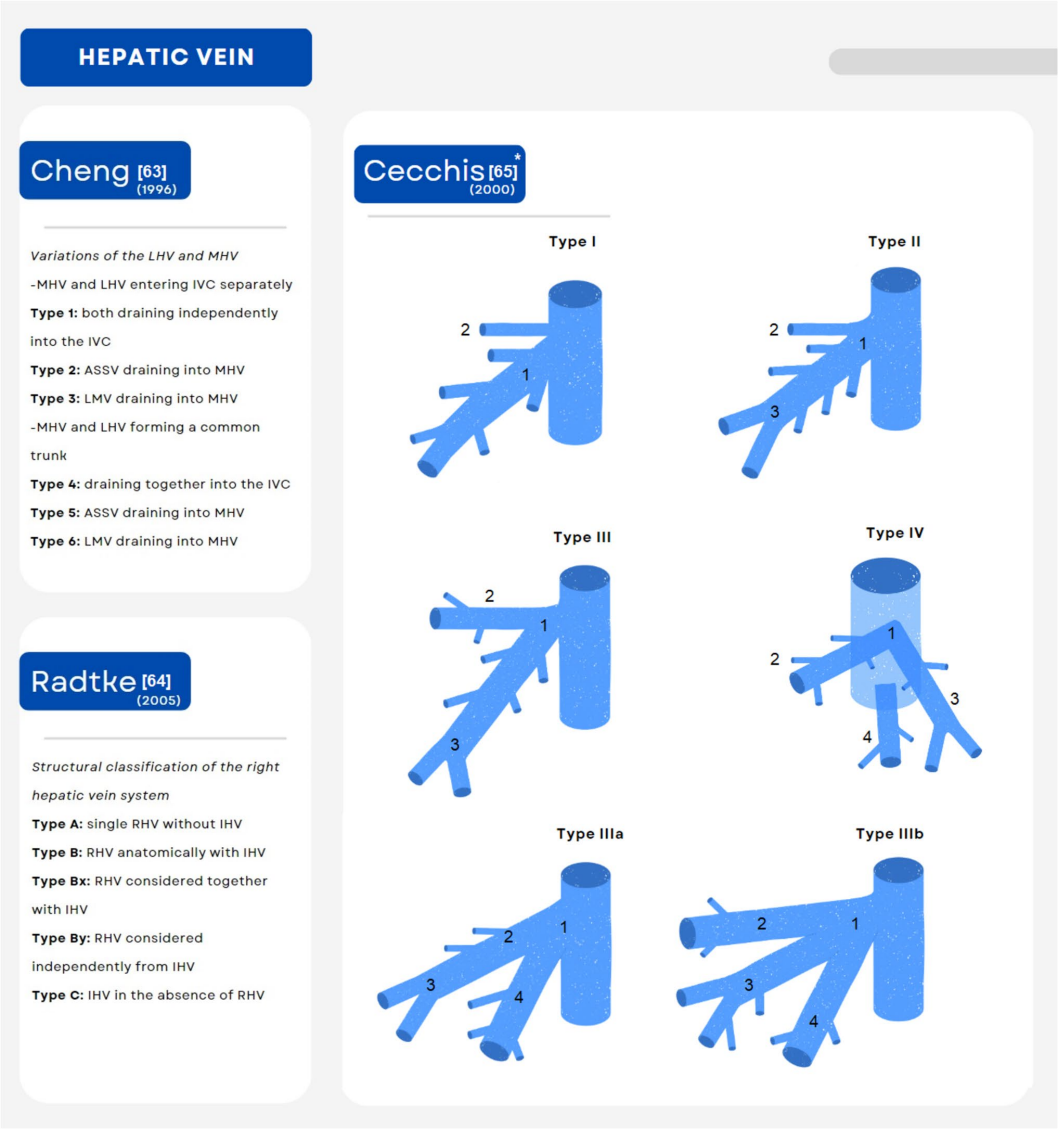
Common/clinically relevant classifications for anatomic variants of the hepatic vein. *ASSV* anterior superior segmental vein, *IHV* inferior hepatic veins, *IVC* inferior vena cava, *LMV* left median vein, *LHV* left hepatic vein, *MHV* middle hepatic vein. *Variations of the superior right hepatic vein [63–65]

The usual anatomic distribution of the HV consist of a right trunk—right hepatic vein (RHV)- which drain segments VII and VI, and a common trunk giving rise to the MHV and left hepatic vein (LHV), draining segments IV, V and VIII, and II-III, respectively [21]. Anatomic variants have been observed in approximately 40% of living donor candidates, with the most common represented by the presence of an accessory (inferior) right hepatic vein draining segment VI and the inferior aspect of segment VII [21]. Relevant classifications for anatomic variants of the hepatic vein are shown in Fig. 3. In a recent study, Senne et al. found that grafts without an accessory RHV demonstrated a pattern of drainage volume distribution of RHV dominance in 82% of the cases, followed by MHV in 16.5% and to a lesser extent LHV (1.5%). On the other hand, in the presence of an accessory HV the dominant drainage volume distribution shifted toward the MHV (49%), followed by the RHV (48%) [29]. In common practice, accessory hepatic veins with small diameter are usually ligated. However, if the diameter is approximately > 4 mm, anastomosis to the recipient inferior vena cava (IVC) is recommended to avoid congestion of the posterior segments [21]. This approach is preferred in our practice. However, practices can vary based on surgeon’s preference. Some groups use the “saline test” or probing, where cold saline is flushed into the vein under evaluation to assess the presence of veno-venous communications and to better guide the need for reconstruction [36, 37].

Despite years of experience and several reports of postoperative outcomes in donors and recipients, inclusion of the MHV in the right graft for LDLT remains a topic of debate. In grafts where the MHV is not procured, identification and characterization of MHV segment V and VIII tributaries requires particular attention. The International Liver Transplant Society Guideline on Living Liver Donation recommends reconstruction of segment V and VIII tributaries with diameters > 5 mm to reduce the risk of anterior segment congestion, graft dysfunction and to improve recipient outcomes [19]. Other practices recommend backtable reconstruction of tributaries measuring ≥ 4 mm [21, 23]. Multiple reconstruction techniques with favorable outcomes have been reported. A common practice to reconstruct the MHV is to create a neo-MHV by end-to-side anastomosis of both tributaries to an interposition graft [38]. Interposition grafts commonly used for reconstruction include cryopreserved deceased donor iliac veins, allogenic vascular grafts and polytetrafluoroethylene grafts [35]. Recent reports support the use of synthetic over biologic grafts for MHV reconstruction in adult-to-adult LDLT [39]. The limited supply, constrained storage life, and restrictions on adequate diameter and length for reconstruction are among the shortcomings of biologic grafts [39]. On the other hand, synthetic grafts may result in accidental migration or persistence of non-degradable foreign bodies [40, 41]. A handful of authors have studied short- and long- term patency rates between different materials for vessel reconstruction [39–41]. However, the impact of the reconstruction material on LDLT recipient’s postoperative outcomes is an understudied area and further research is needed to better elucidate the most suitable material for MHV reconstruction in LDLT.

Finally, identification of recipients and donors’ factors associated with negative impact on outflow hemodynamics is critical during LDLT pre-operative evaluation. Poorer outcomes, including development of small for size syndrome, are associated to recipients with a graft-to-recipient weight ratio (GRWR) < 0.8%, presence of portal hypertension, sarcopenia and high Model for end-stage Liver disease (MELD) scores [35]. Similarly, grafts from donors with steatosis, which limits tolerance to portal hyperperfusion, as well as those with low-estimated future remnant volume (FRV) conveys increased risks for negative outcomes [35]. Therefore, presence of these conditions require special attention and reconsideration of donor-recipient pairing might be needed.

### Biliary anatomy

**Fig. 4:**
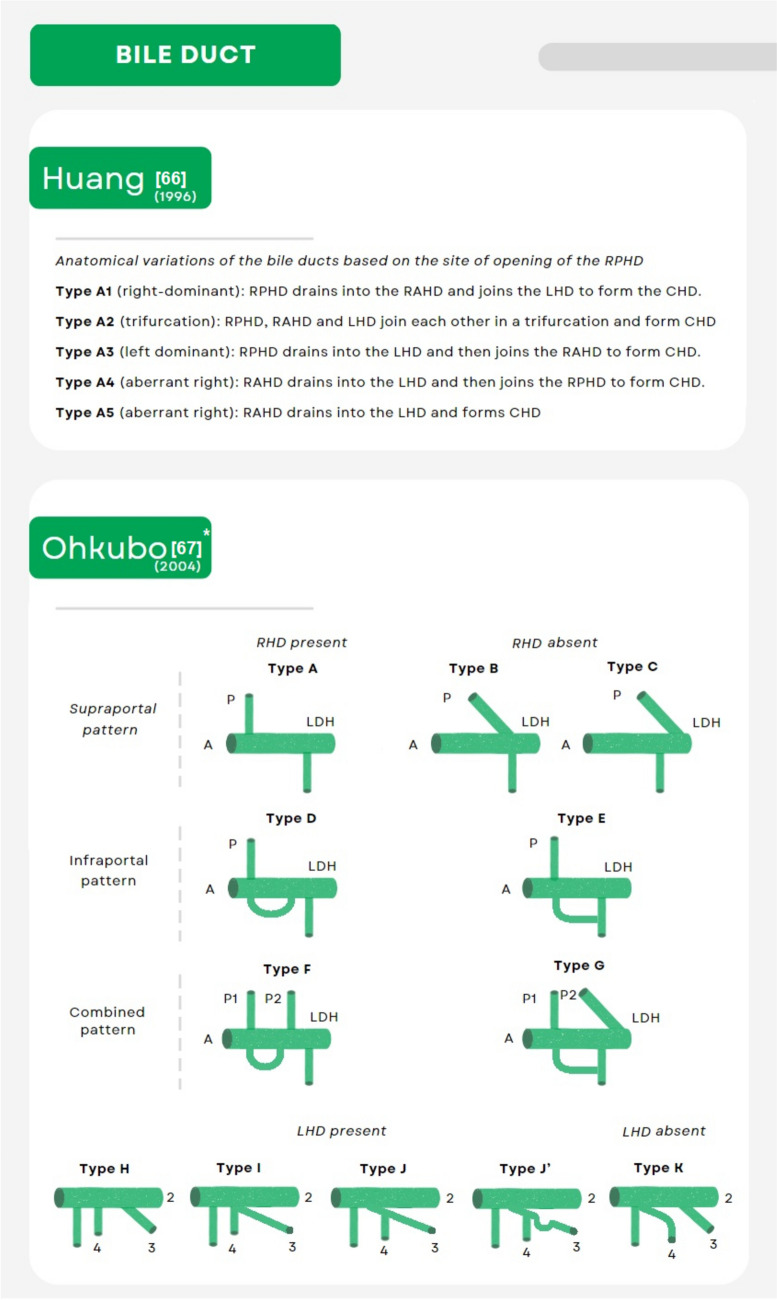
Common/clinically relevant classifications for anatomic variants of the bile ducts. *A* anterior-sectional bile duct, *CHD* common hepatic duct, *LHD* left hepatic duct, *RAHD* right-anterior hepatic duct, *RPHD* right-posterior hepatic duct, *P* posterior-sectional bile duct, *Confluence patterns of the right and left intrahepatic bile ducts [66, 67]

**Fig. 5:**
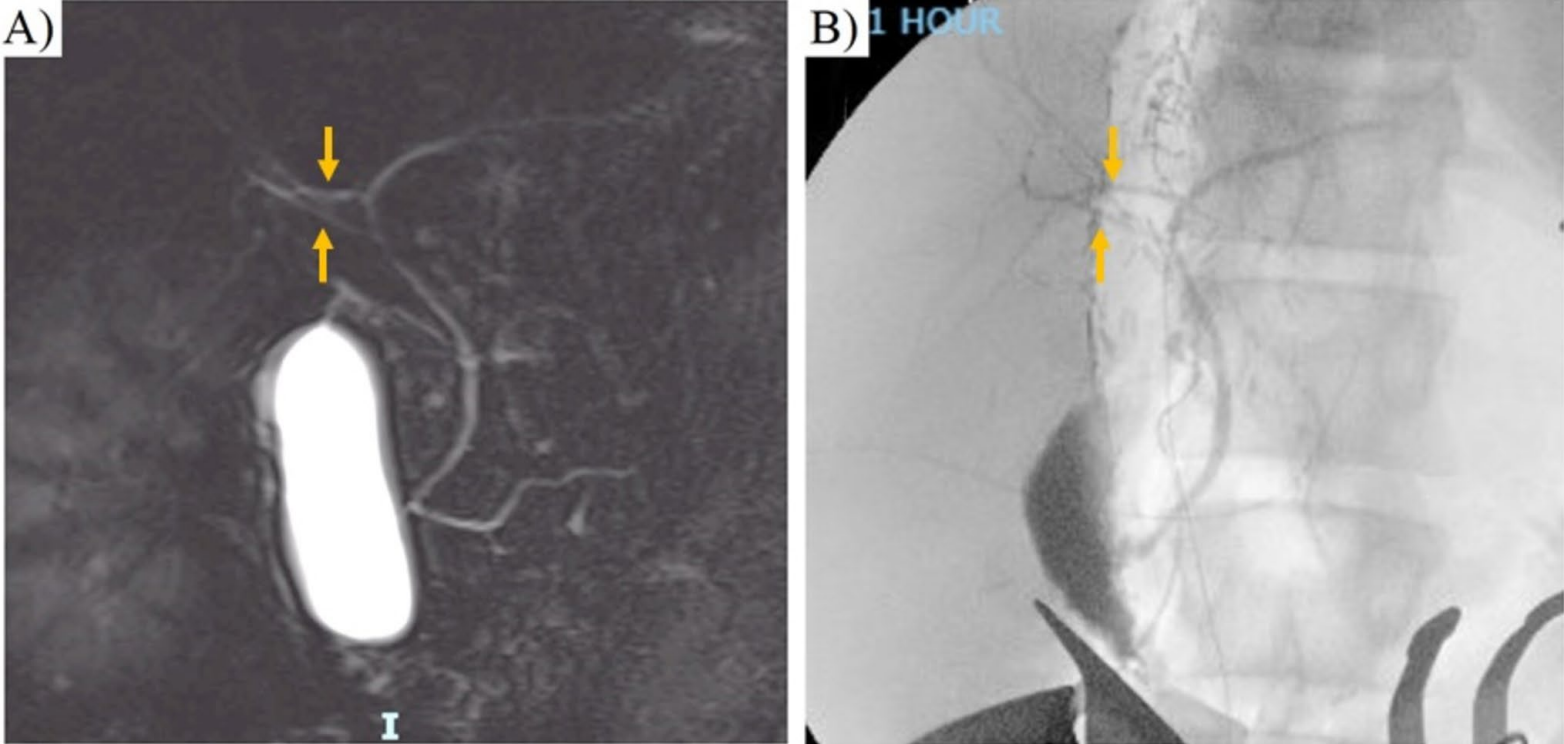
Pre-operative assessment with magnetic resonance cholangiopancreatography (MRCP) in a living donor candidate with two separate right hepatic ducts (**A**). Intraoperative cholangiogram of the same donor consistent with preoperative MRI findings with evidence of two right hepatic ducts draining separately into the main and left hepatic ducts (**B**). Arrows: right-anterior and right-posterior hepatic ducts

The intricate anatomy of bile duct confluence adds complexity to the already technically challenging donor and recipient surgery. Moreover, surgeons must be aware of the different possible anatomic variants (Fig. 4), which can be found in almost half of the population [21, 42]. Importantly, anatomic variations are more common in the right hepatic duct, including drainage of the right-posterior duct into the left hepatic duct, trifurcation of the biliary ducts and right-posterior duct draining into the common hepatic duct [21–42]. Therefore, detailed evaluation of donors’ bile duct anatomy is needed to achieve successful postoperative outcomes. (Fig. 5).

Intraoperative cholangiogram with cannulation of the cystic duct is the gold standard imaging technique for evaluation of biliary anatomy [23, 42]. Nonetheless, pre-operative assessment with magnetic resonance cholangiopancreatography (MRCP) is usually the preferred method for pre-operative donor evaluation due to its non-invasive nature, high sensitivity and high accuracy in detecting anatomic variants in the biliary tract [21, 42]. Diagnostic accuracy, possibility to detect second and third order bile ducts, and degree of compromise by respiratory motion and ghosting artifacts varies among techniques [21, 23]. Data suggest that a combination of T1-weighted and T2-weighted sequences for depiction of bile ducts provides improved diagnostic accuracy [21, 23]. As for the hepatobiliary contrast agent, gadoxetate disodium is usually preferred due to its higher percentage of biliary excretion and shorter delay for the hepatobiliary phase when compared to other agents [21]. In the last decade, the potential benefits of near-infrared imaging with indocyanine green (ICG), a sterile, liver-specific, non-ionizing, non-toxic and water-soluble fluorescent dye as an adjuvant in hepatobiliary surgery has been widely recognized [43, 44]. Initial experience with ICG fluorescence cholangiography for living donor hepatectomy demonstrated its role in improving outcomes for LDLT, achieving reduction of biliary complications for donors and recipients [45, 46]. The real-time identification of the biliary tract around the hilar plate allows for optimal identification of bile duct anatomy variations and facilitate it safe dissection and appropriate division points for bile ducts during open or laparoscopic LDLT [47]. Current consensus recommendations for the use of ICG fluorescence in hepatobiliary surgery recommends a dose of 2.5 mg ICG administered intravenously for LDLT biliary mapping, 15 to 30 min before dissection of the hilar plate [47]. Nonetheless, available literature pertinent to ICG fluorescence cholangiography for living donor hepatectomy is limited. Current practices support its use as a complementary tool, rather than a replacement for the current gold standard. Further evidence is needed in order standardize practices around its use, particularly under emerging approaches for living donor hepatectomy such minimally invasive techniques.

Although, a comprehensive pre-operative imaging of donors’ biliary anatomy will not ensure a complication-free procedure, it can definitely aid to minimize the associated risks. Biliary complications occur in up to 9% of donors and represent an important cause of donor morbidity following living donor hepatectomy [15, 48]. The severity of the complications in donors ranges from minor and self-limited to severe complications—usually related to bile leaks and biliary fistulas—leading to longer length of stay, higher readmission rates, higher costs of care, need for repeated interventions and lower quality of life [15, 48, 49]. Risk factors associated with biliary complications include anatomic intrahepatic duct type, donation of the right lobe and number of bile duct orifices in the RL [48].

Decision to proceed with donation in cases with complex biliary anatomy requires considerate deliberation based on centers’ capability and surgeons’ experience. Some suggest that donors with 3 or more right hepatic ducts, as well as those with variations that could compromise segmental bile drainage should be avoided [50]. Therefore, pre-operative mapping of the biliary anatomy is an important step during surgical planning, allowing the surgeon to determine donor candidacy, the most adequate bile duct transection line and most suitable technique for biliary anastomosis [23]. In the recipient, single duct anastomosis are more common, and are usually performed by duct-to-duct anastomosis with or without ductoplasty or Roux-en-Y. Nonetheless, presence of more than one duct constitutes an important number of cases and require particular attention to avoid technical complications and negative postoperative outcomes [51]. Decision to perform biliary reconstructions or ligation in such cases remains a topic of debate.

### Liver volumes

The regenerative ability of the liver into a functional unit is influenced by the donor remnant fraction [12]. Although, there is not a standardized cut-off value, common practice is to ensure a remnant liver volume (RLV) to total liver volume (TLV) ratio ≥ 30% [52]. A recent meta-analysis found overall and minor morbidity to be significantly lower among donors with RLV/TLV ≥ 30% when compared to those < 30%. In addition, donors with RLV/TLV < 30% demonstrated a higher degree of impartment in postoperative liver function, evidenced by significantly higher peak bilirubin and peak INR levels vs donors in the RLV/TLV ≥ 30% group [52]. Therefore, living donation from donors with a low RLV/TLV (< 30%) may induce liver dysfunction and should be avoided if possible. Although reported incidence of post-hepatectomy liver failure is variable, it can be as high as 32% [21]. Therefore, estimation of liver volume is vital to ensure donor safety as the primary outcome during LDLT. However, when deciding appropriate donor-recipient pairing, it is important to have in mind that a GRWR ≥ 0.8% safeguards favorable outcomes in the recipient [53].

**Fig. 6:**
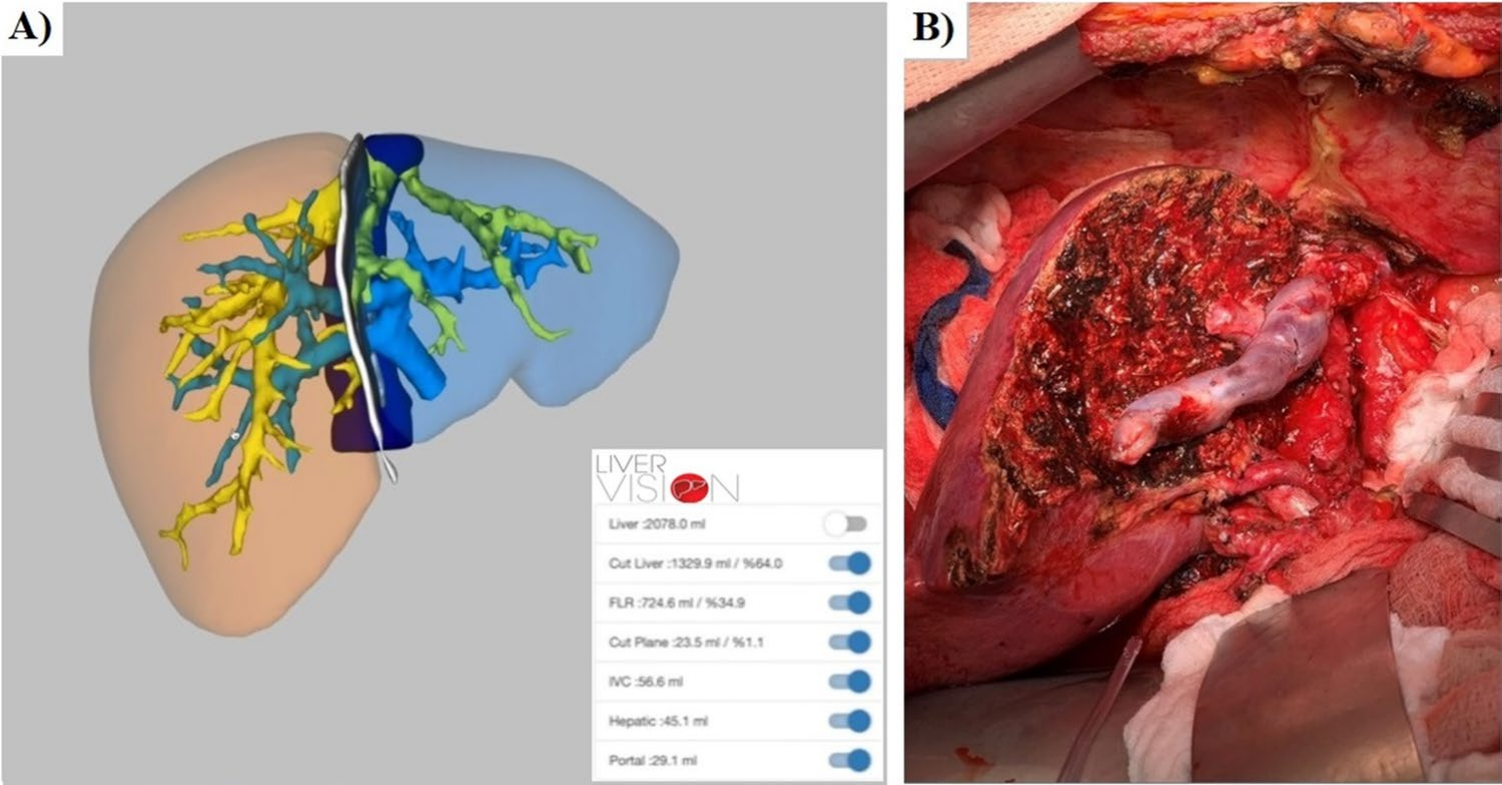
Liver volumetric assessment in a living donor candidate performed using semi-automated liver volumetry software with 3D reconstructions and segmentation of the liver (LiverVision® software) (**A**), allowing safe practices and accurate prediction of the actual graft weight for living donor liver transplantation (**B**)

Precise estimation of liver volume is challenging, as it is affected not only by the technique used, but also by several donor’s characteristics including age, gender and presence of steatosis [21, 53]. Data suggest that pre-operative volume estimations can vary up to 10% when compared to actual graft weight measured intraoperatively [53]. Volume estimation can be performed by manual techniques including calculations based on surface body area, as well as semi-automated (conventional CT or MRI volumetry) and fully-automated imaging techniques with 3D reconstructions and segmentation of the liver [21, 53–55]. (Fig. 6). Technical advances and better understanding of deep learning models have allowed for the development of automatic methods that are reliable, operator independent, can be used in parallel and can provide improved performance of segmentation [54]. Moreover, conventional 2D imaging techniques fall short on consideration of blood volume within the liver, while modern 3D techniques with novel software allows for exclusion of blood volume, providing a more accurate assessment of liver parenchyma and less overall error in volume estimation [53, 55]. Efforts to develop highly accurate and advanced software specifically designed for the LDLT setting are currently ongoing [56, 57]. A recent report demonstrated accurate prediction of the actual graft weight among 106 living donors undergoing right lobe hepatectomy, left lobe hepatectomy and left lateral segmentectomies using a novel software. [56] In addition, it was found that 95.3% of the measurements were within the 95% range of agreement between the software and established manual measurements [56]. Development of LDLT-specific validated software such as that one is an important pillar toward safer practices in LDLT. Nonetheless, regardless of the technique used, to ensure donor safety surgeons need to account for all the possible factors leading to overestimation and underestimation errors on a case-by-case basis when deciding donor candidacy.

## Conclusion

Donor safety remains the main priority in LDLT. Anatomical variants in the vascular and biliary system are common among healthy donors. Although only in limited cases such variants affect donor candidacy, failure to identify them jeopardize donor and recipient safety, and increases technical complexity to the case. Experience suggests that maximizing donor safety and lessening their risks is a result of integrated experience between hepatobiliary and transplant surgery, along with multidisciplinary efforts in performing a comprehensive pre-operative donor assessment. Although, technical advances have increased the accuracy of volume estimation, over- or under-estimation remains a challenge that needs further attention.

## Data Availability

Data sharing not applicable to this article as no datasets were generated or analyzed during the current study.
